# Tuberculous/BCG‐Related Chronic Orchiepididymitis With Fistulization: Expanded Case Report and Literature Review

**DOI:** 10.1155/criu/2998895

**Published:** 2026-04-27

**Authors:** Guilherme Bernardo, João A. Carvalho, Maria Alzamora, João Lobo, António Morais

**Affiliations:** ^1^ Department of Urology, ULS Amadora Sintra, Amadora, Portugal; ^2^ Department of Urology, Portuguese Oncology Institute of Porto (IPO Porto), Porto, Portugal; ^3^ Deparment of Pathology, Portuguese Oncology Institute of Porto (IPO Porto), Porto, Portugal; ^4^ Department of Cancer Biology and Epigenetics Group, Research Center of IPO Porto CI-IPOP/CI-IPOP@RISE Health Research Network, Portuguese Oncology Institute of Porto (IPO Porto/Porto Comprehensive Cancer Center Raquel Seruca), Porto, Portugal; ^5^ Department of Pathology and Molecular Immunology, School of Medicine and Biomedical Sciences—University of Porto (ICBAS-UP), Porto, Portugal

**Keywords:** BCG therapy, genitourinary tuberculosis, granulomatous inflammation, intravesical immunotherapy, orchiepididymitis

## Abstract

Intravesical Bacillus Calmette–Guérin (BCG) therapy is a well‐established treatment for high‐grade nonmuscle invasive bladder cancer. However, infectious complications such as granulomatous orchiepididymitis may rarely occur. We report a case of chronic necrotizing granulomatous orchiepididymitis with fistulization following intravesical BCG instillations. The patient presented with persistent epididymal swelling, caseous discharge, and inadequate response to conventional antibiotic therapy. Polymerase chain reaction (PCR) testing identified *Mycobacterium tuberculosis* complex. Despite clinical improvement under antituberculous therapy, surgical orchiectomy was required due to persistent anatomical destruction. Histopathological examination confirmed necrotizing granulomas with Langhans‐type giant cells, consistent with bacillary orchiepididymitis.

## 1. Introduction

Intravesical Bacillus Calmette–Guérin (BCG) therapy is an established treatment for high‐grade nonmuscle invasive bladder cancer, significantly reducing recurrence and progression rates [1]. Although generally safe, BCG instillation may be associated with local and systemic infectious complications, in approximately 1%–5% of patients [2, 3]. Genitourinary complications such as granulomatous orchiepididymitis are rare, with reported incidence below 0.5%. The pathogenesis is not fully understood, but retrograde spread through the vas deferens is considered the most likely mechanism, whereas hematogenous dissemination may occur in selected cases. Differentiating BCG infection from *Mycobacterium tuberculosis* remains challenging. Clinical presentation is often nonspecific, and microbiological confirmation may be challenging due to the paucibacillary nature of the disease. We report a case of chronic necrotizing granulomatous orchiepididymitis with fistulization following intravesical BCG therapy.

## 2. Case Presentation

A 69‐year‐old man with a history of high‐grade pT1 nonmuscle invasive bladder carcinoma underwent transurethral resection followed by intravesical BCG therapy. He completed induction therapy and developed symptoms during early maintenance therapy, shortly after the second cycle of instillations.

He presented with a progressive epididymal swelling, tenderness, and later spontaneous fistulization with caseous discharge. Conventional antibiotic therapy (fluoroquinolones) offered no improvement.

Scrotal ultrasonography demonstrated heterogeneous enlargement of the epididymis with hypoechoic areas suggestive of abscess formation, associated hypervascularity on Doppler imaging, and a sinus tract extending toward the scrotal skin. No suspicious solid intratesticular mass was identified.

Microbiological investigation revealed positivity for *M. tuberculosis* complex on PCR from caseous material. Auramine stain was positive, whereas Ziehl–Neelsen staining on tissue samples following orchiectomy was negative (Table 1).

**Table 1:** Microbiological and laboratory findings supporting the diagnosis of mycobacterial orchiepididymitis.

Test	Result	Reference/interpretation
PCR—*Mycobacterium tuberculosis* complex	Positive	Negative
Auramine stain	Positive	Negative
Ziehl–Neelsen stain (tissue)	Negative	Negative

The patient was evaluated for systemic tuberculosis. Chest X‐ray showed no evidence of pulmonary involvement, and no systemic symptoms were present.

**Figure 1:**
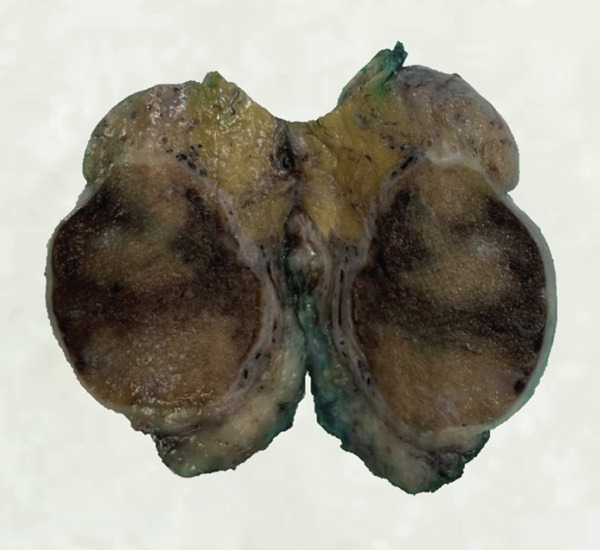
Orchidectomy specimen demonstrating extensive destruction of the epididymis and testicular involvement.

A standard antituberculous regimen (HRZE) was initiated. Partial clinical improvement was observed; however, persistent fistulization led to left orchiectomy (Figure 1).

**Figure 2:**
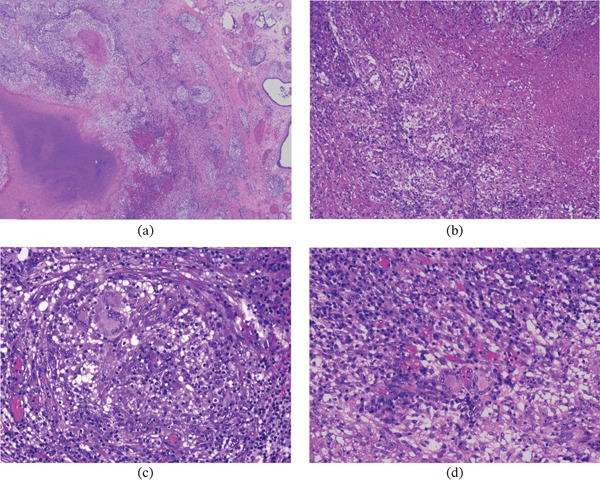
Representative hematoxylin and eosin–stained sections showing necrotizing granulomatous inflammation consistent with bacillary orchiepididymitis. (a) Low‐power view (×20) displaying extensive granulomatous inflammation with necrosis involving predominantly the epididymis. (b) Intermediate magnification (×100) highlighting associated interstitial changes, including fibrosis and hemorrhage within the inflamed tissue. (c–d) Higher magnification (×200) demonstrating a dense lymphoplasmacytic inflammatory infiltrate with numerous multinucleated giant cells.

Histopathological examination revealed a chronic necrotizing granulomatous inflammatory process with multinucleated giant cells and abundant lymphoplasmacytic infiltrate, consistent with granulomatous orchiepididymitis suggestive of mycobacterial infection (Figure 2).

## 3. Discussion

A key diagnostic challenge in this case is the distinction between BCG‐related infection (*Mycobacterium bovis* BCG) and true *M. tuberculosis* infection. PCR assays detecting *M. tuberculosis* complex often lack species‐level discrimination. The absence of pulmonary findings supports a localized genitourinary process, favoring a BCG‐related complication, although true tuberculosis cannot be fully excluded.

Although intravesical BCG has an excellent oncological safety profile, mycobacterial dissemination may occur, leading to a localized or systemic infection. Several pathobiological mechanisms have been proposed. The most widely accepted theory is retrograde spread of mycobacteria from the lower urinary tract through the vas deferens to the epididymis [4, 5]. This hypothesis is supported by the frequent initial involvement of the epididymal tail, close temporal relation with BCG instillations, and absence of pulmonary or disseminated disease in most cases. Hematogenous dissemination represents an alternative mechanism, particularly in patients with delayed presentation, bilateral disease, or systemic symptoms [6, 7]. This pathway is also considered when molecular techniques detect *M. tuberculosis* complex rather than *M. bovis* BCG, although routine PCR assays often lack species‐level discrimination [8, 9]. Clinically, patients typically present with scrotal swelling, pain, induration, or nodularity, frequently mimicking bacterial epididymo‐orchitis or testicular neoplasia [10, 11]. Imaging findings are nonspecific, often showing heterogeneous epididymal enlargement or abscess formation, contributing to diagnostic delay [12]. Diagnosis is challenging due to the paucibacillary nature of the disease. Acid‐fast bacilli staining is often negative, and cultures require prolonged incubation with limited sensitivity [13]. PCR‐based techniques improve diagnostic yield but may not distinguish between BCG and wild‐type tuberculosis without targeted assays [13, 14]. Antituberculous therapy remains the cornerstone of management. Surgical intervention is indicated in cases with abscess formation, fistulization, persistent symptoms, or irreversible anatomical destruction, as observed in the present case [15].

## 4. Conclusions

BCG‐related granulomatous orchiepididymitis is an uncommon but clinically significant complication of intravesical immunotherapy. A high index of suspicion, appropriate microbiological testing, and timely surgical intervention are essential for optimal outcomes.

## Author Contributions

Guilherme Bernardo had full access to all data and takes responsibility for data integrity and analysis accuracy. Concept and design: Guilherme Bernardo, Maria Alzamora, João A. Carvalho, and António Morais. Acquisition, analysis, or interpretation of data: Guilherme Bernardo, Maria Alzamora, João Lobo, João A. Carvalho, and António Morais. Drafting of the manuscript: Guilherme Bernardo, Maria Alzamora, João A. Carvalho, and António Morais. Critical review of the manuscript for important intellectual content: Guilherme Bernardo, Maria Alzamora, João Lobo, João A. Carvalho, and António Morais. Supervision: João Lobo, João A. Carvalho, and António Morais.

## Funding

No funding was received for this manuscript.

## Disclosure

All authors have reviewed the final version to be published and agreed to be accountable for all aspects of the work.

## Consent

Informed consent for treatment and open access publication was obtained or waived by all participants in this study.

## Conflicts of Interest

The authors declare no conflicts of interest.

## Data Availability

The data that support the findings of this study are available on request from the corresponding author. The data are not publicly available due to privacy or ethical restrictions.

## References

[1] Lamm D. L. , van der Meijden P. M. , Morales A. , Brosman S. A. , Catalona W. J. , Herr H. W. , Soloway M. S. , Steg A. , and Debruyne F. M. J. , Incidence and Treatment of Complications of Bacillus Calmette-Guerin Intravesical Therapy in Superficial Bladder Cancer, Journal of Urology. (1992) 147, 3 Part 1, 596–600. 1538436 10.1016/s0022-5347(17)37316-0

[2] Böhle A. and Bock P. R. , Intravesical Bacille Calmette-Guérin Versus Mitomycin C in Superficial Bladder Cancer: Formal Meta-Analysis of Comparative Studies on Recurrence and Toxicity, European Urology. (2004) 46, 401–409, 10.1016/j.urology.2003.11.049, 2-s2.0-1842789737.

[3] Pérez-Jacoiste Asín M. A. , Fernández-Ruiz M. , López-Medrano F. et al., Bacillus Calmette-Guérin (BCG) Infection Following Intravesical BCG Administration as Adjunctive Therapy for Bladder Cancer, Medicine.(2014) 93, 10.1097/md.0000000000000119, 2-s2.0-84922481923. PMC4602419 25398060

[4] Golden M. P. and Vikram H. R. , Extrapulmonary Tuberculosis: An Overview, American Family Physician. (2005) 72, 1761–1768, 10.1016/b978-1-4160-3988-4.00034-2. 16300038

[5] Pai M. , Behr M. A. , Dowdy D. et al., Tuberculosis, Lancet. (2016) 387, 1211–1226, 10.1016/s0140-6736(15)00151-8, 2-s2.0-84961214191. 26377143 PMC11268880

[6] Jacobsen F. , Holzer M. , and Moter A. , Diagnostic Pitfalls in Mycobacterial Infections Following Intravesical BCG Therapy, Infection. (2018) 46, 695–703, 10.2165/00128415-199304350-00016.

[7] García-Rodríguez J. F. and Álvarez-Díaz H. , Genitourinary Tuberculosis: Clinical Manifestations and Diagnosis, Clinical Microbiology Reviews Journal. (2021) 34, 00009–00020, 10.1007/s10096-018-3408-2, 2-s2.0-85055879708.

[8] Ojea A. , Nogueira J. L. , Solsona E. , Flores N. , Gómez J. M. , Molina J. R. , Chantada V. , Camacho J. E. , Piñeiro L. M. , Rodríguez R. H. , Isorna S. , Blas M. , Martínez-Piñeiro J. A. , Madero R. , and CUETO Group (Club Urológico Español De Tratamiento Oncológico) , A Multicentre, randomised Prospective Trial Comparing Three Intravesical Adjuvant Therapies for Intermediate-Risk Superficial Bladder cancer: low-dose bacillus Calmette-Guerin (27 mg) versus very low-dose bacillus Calmette-Guerin (13.5 mg) versus mitomycin C, Journal of Urology. (2007) 52, no. 5, 1398–1406, 10.1016/j.eururo.2007.04.062, 2-s2.0-34548859502, 17485161. 17485161

[9] Kamat A. M. , Shore N. , Hahn N. , Sylvester R. J. , Böhle A. , Palou J. , Lamm D. L. , Brausi M. , Soloway M. , Persad R. , Buckley R. , Colombel M. , and Witjes J. A. , Definitions, Endpoints, and Clinical Trial Designs for Non-Muscle-Invasive Bladder Cancer, Nature Reviews Urology. (2016) 13, 341–353, 10.1038/s41585-023-00789-0.

[10] Brausi M. , Oddens J. , Sylvester R. , Bono A. , van de Beek C. , van Andel G. , Gontero P. , Turkeri L. , Marreaud S. , Collette S. , and Oosterlinck W. , Side Effects of Bacillus Calmette-Guérin Therapy in the Treatment of Intermediate- and High-Risk Ta, T1 Papillary Carcinoma of the Bladder, European Urology. (2012) 62, 58–64, 10.1016/j.eururo.2010.02.037, 2-s2.0-77951644129. 23910233

[11] Kaasinen E. , Complications of Intravesical Bacillus Calmette-Guérin Therapy, Scandinavian Journal of Infectious Diseases. (2002) 34, 573–576, 10.1159/000471561, 2-s2.0-0024390186.

[12] Zignol M. , Dean A. S. , Falzon D. , van Gemert W. , Wright A. , van Deun A. , Portaels F. , Laszlo A. , Espinal M. A. , Pablos-Méndez A. , Bloom A. , Aziz M. A. , Weyer K. , Jaramillo E. , Nunn P. , Floyd K. , and Raviglione M. C. , Twenty Years of Global Surveillance of Antituberculosis-Drug Resistance, Lancet Infectious Diseases. (2016) 375, no. 11, 1081–1089, 10.1056/nejmsr1512438, 2-s2.0-84988353653, 27626523. 27626523

[13] Witjes J. A. , Bruins H. M. , and Cathomas R. , European Association of Urology Guidelines on Non-Muscle.

[14] Paner G. P. , Montironi R. , Re J. , Witjes A. , Bruins H. M. , and Cathomas R. , Invasive Bladder Cancer (TaT1 and CIS), European Urology. (2021) 79, 82–104, 10.1016/j.eururo.2020.08.014. 32873442

[15] Figueiredo A. A. , Lucon A. M. , and Srougi M. , Surgical Management of Genitourinary Tuberculosis, Nature Clinical Practice Urology. (2008) 10, 207–217, 10.1038/ncpuro1148, 2-s2.0-49249104589.

